# Brachyury identifies a class of enteroendocrine cells in normal human intestinal crypts and colorectal cancer

**DOI:** 10.18632/oncotarget.7202

**Published:** 2016-02-05

**Authors:** Jana Jezkova, Jason S. Williams, Filipe Pinto, Stephen J. Sammut, Geraint T. Williams, Simon Gollins, Ramsay J. McFarlane, Rui Manuel Reis, Jane A. Wakeman

**Affiliations:** ^1^ North West Cancer Research Institute, School of Medical Sciences, Bangor University, Bangor, UK; ^2^ Life and Health Sciences Research Institute (ICVS), School Health Sciences, University Minho, Braga, Portugal; ^3^ ICVS/3B's - PT Government Associate Laboratory, Braga/Guimarães, Portugal; ^4^ Institute of Cancer and Genetics, Cardiff University Medical School, Cardiff, UK; ^5^ North Wales Cancer Treatment Centre, Betsi Cadwaladr University Health Board, Bodelwyddan, UK; ^6^ NISCHR Cancer Genetics Biomedical Research Unit, Cardiff, UK; ^7^ Molecular Oncology Research Center, Barretos Cancer Hospital, Barretos, SP, Brazil

**Keywords:** Brachyury, enteroendocrine cells, small intestine/colon, crypts, colorectal cancer

## Abstract

Normal homeostasis of adult intestinal epithelium and repair following tissue damage is maintained by a balance of stem and differentiated cells, many of which are still only poorly characterised. Enteroendocrine cells of the gut are a small population of differentiated, secretory cells that are critical for integrating nutrient sensing with metabolic responses, dispersed amongst other epithelial cells. Recent evidence suggests that sub-sets of secretory enteroendocrine cells can act as reserve stem cells. Given the link between cells with stem-like properties and cancer, it is important that we identify factors that might provide a bridge between the two. Here, we identify a sub-set of chromogranin A-positive enteroendocrine cells that are positive for the developmental and cancer-associated transcription factor Brachyury in normal human small intestinal and colonic crypts. Whilst chromogranin A-positive enteroendocrine cells are also Brachyury-positive in colorectal tumours, expression of Brachyury becomes more diffuse in these samples, suggesting a more widespread function in cancer. The finding of the developmental transcription factor Brachyury in normal adult human intestinal crypts may extend the functional complexity of enteroendocrine cells and serves as a platform for assessment of the molecular processes of intestinal homeostasis that underpins our understanding of human health, cancer and aging.

## INTRODUCTION

The adult intestinal epithelium is one of the most rapidly self-renewing tissues of the body, giving rise to differentiated cells for digestion and absorption of food. Constant insult from luminal contents in the gut necessitates continuous turnover and renewal of differentiated epithelial cells that carry out homeostatic functions, including cells of the absorptive and secretory lineages. Whilst rare (∼1% of crypt cells), enteroendocrine cells (EECs), which can be divided into specific EEC sub-groups, are central to this process through their secretion of hormones [1]. Continuous replacement of differentiated cells is brought about by multipotent stem cells (SCs), which predominantly reside in the crypt-base [Lgr5-marked crypt-base columnar cells], and their proliferating progenitor-derivatives [transient amplifying (TA) cells] [2, 3]. However, the adult intestinal SC compartment is more complex in nature, additionally comprising of poorly understood sub-types of SCs, some rapidly cycling, others quiescent and more damage resistant (e.g., Bmi1/Hopx/Tert/Lrig1-marked). One function for the additional sub-types is that they act as reserves that can replace rapidly cycling Lgr5-SCs after injury or with age [4]. The plasticity of these cells in response to various cues is unclear; cell types have been identified with SC capacity which are derived from progenitors or differentiated cell types. For example, a sub-set of post-mitotic (non-proliferating) EECs [marked as Chromogranin A (ChgA)-positive/Ki67-negative] has been found at the crypt-base of the mouse small intestine and these cells demonstrate SC markers such as Lgr5 [5]. Furthermore, the transcription factor Sox9 is present in the SC/progenitor zone of the mouse small intestine where it regulates proliferation and differentiation in a dose-dependent manner; Lgr5-SCs are Sox9-low, but a population of differentiated EECs expressing ChgA shows high levels of Sox9. This latter population is Ki67-negative (therefore post-mitotic) but does have regenerative capacity following radiation damage [6, 7].

It is possible that different sub-sets of reserve SCs exist in the intestinal crypt, determined by the presence of specific transcription factors such as Sox9 and others, not yet determined. The importance of EECs to crypt homeostasis is further highlighted by recent studies which show that intestinal SC divisions in *Drosophila* mid-gut are stimulated by EECs [8]. Taken together, sub-groups of EECs might play diverse and critical functions in normal gut homeostasis, in regeneration following damage and during aging.

The link between stem and progenitor cells and colorectal cancer (CRC) is established [9, 10], and like normal intestinal epithelium, cancers consist of a heterogeneous mix of cells including stem-like cells that are the source of cells within the tumour. Stem-like cancer cells (CSCs) in CRC are marked by Lgr5 [11] and such Lgr5-marked CSCs are present at high levels in human adenomas and invading cells [12]. CRCs have also been shown to contain ChgA-positive endocrine cells and a worse prognosis may be associated with endocrine differentiation [13]. Furthermore, neuroendocrine differentiation in CRCs has been highlighted as an independent prognostic factor in stage III–IV disease [14].

Brachyury is a developmentally regulated T-box transcription factor involved in controlling cell movements and differentiation [15]. Outside its developmental context, aberrant expression of Brachyury is associated with a number of solid tumour types, including CRC [16, 17], cancer SCs [18] and also poor prognosis [19]. Brachyury was also shown to be necessary for spermatogonial SC fate in adult mouse testis where it is critically required for germline SC maintenance and self-renewal [20].

We show here that the developmental and cancer-associated transcription factor Brachyury identifies a sub-population of EECs in normal adult human intestinal crypts, adding further depth and complexity to the function of this group of cells in adult intestinal crypts. Furthermore, Brachyury continues to mark this group of EECs in colorectal tumours, but its expression becomes more widely distributed, suggesting a more diverse function in cancer.

## RESULTS

### Brachyury is expressed in adult, normal intestinal tissue

**Figure 1:**
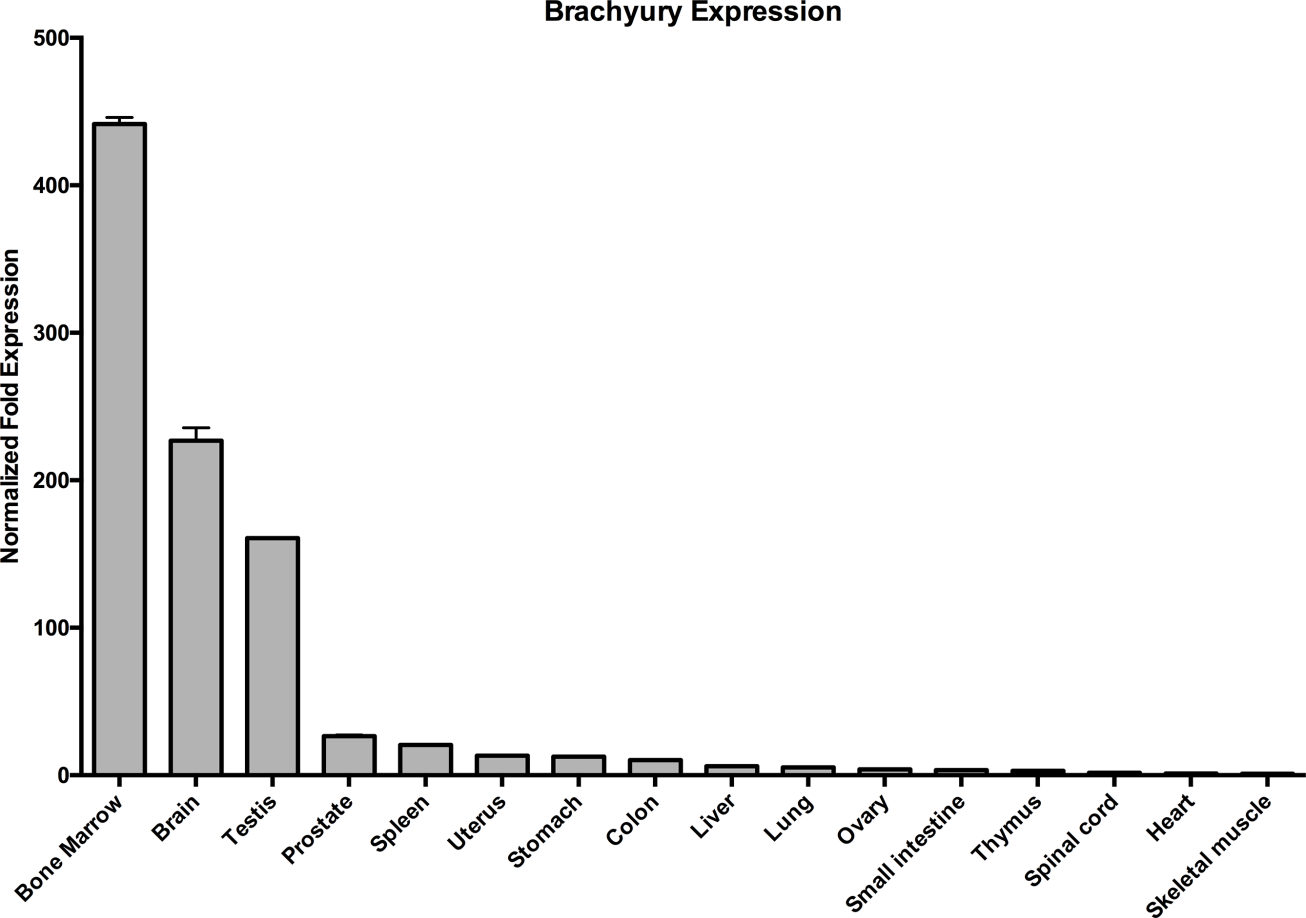
Brachyury is present in normal adult colon and small intestine qRT-PCR analysis of *Brachyury* in normal human tissues showing low level expression in colon and small intestine. Results were normalised to a combination of two endogenous reference genes (*GAPDH/HSPCB*). ‘Normalised Fold Change’ represents gene expression relative to the lowest value observed i.e. skeletal muscle which is set at ‘1’. Error bars show standard error of the mean.

Given the function of Brachyury in SCs of mouse testis and its association with cancer, we wanted to confirm the expression profile of *Brachyury* in normal adult human tissues, including the testis, and whether it exhibited a Cancer Testis Antigen-gene (CTA-gene)-like profile; that is, expressed in only normal adult immune-privileged tissues (i.e. testis and brain) and aberrantly in cancers [21]. We therefore performed qRT-PCR analysis of *Brachyury* on a range of normal tissues (Figure 1). The expression pattern observed loosely follows a CTA/central nervous system gene expression profile (with the exception of SC-rich bone marrow), that is, high levels in testis and brain of normal tissues. However, we also observed low level *Brachyury* expression in the colon and small intestine (amongst other tissues), extending existing studies [22] (see also Supplementary Figures 1A, 2B, 3C) suggesting a wider function for Brachyury in a more extensive group of adult somatic cells.

### Brachyury marks a sub-group of non-proliferating ChgA-EECs in adult intestinal crypts

**Figure 2:**
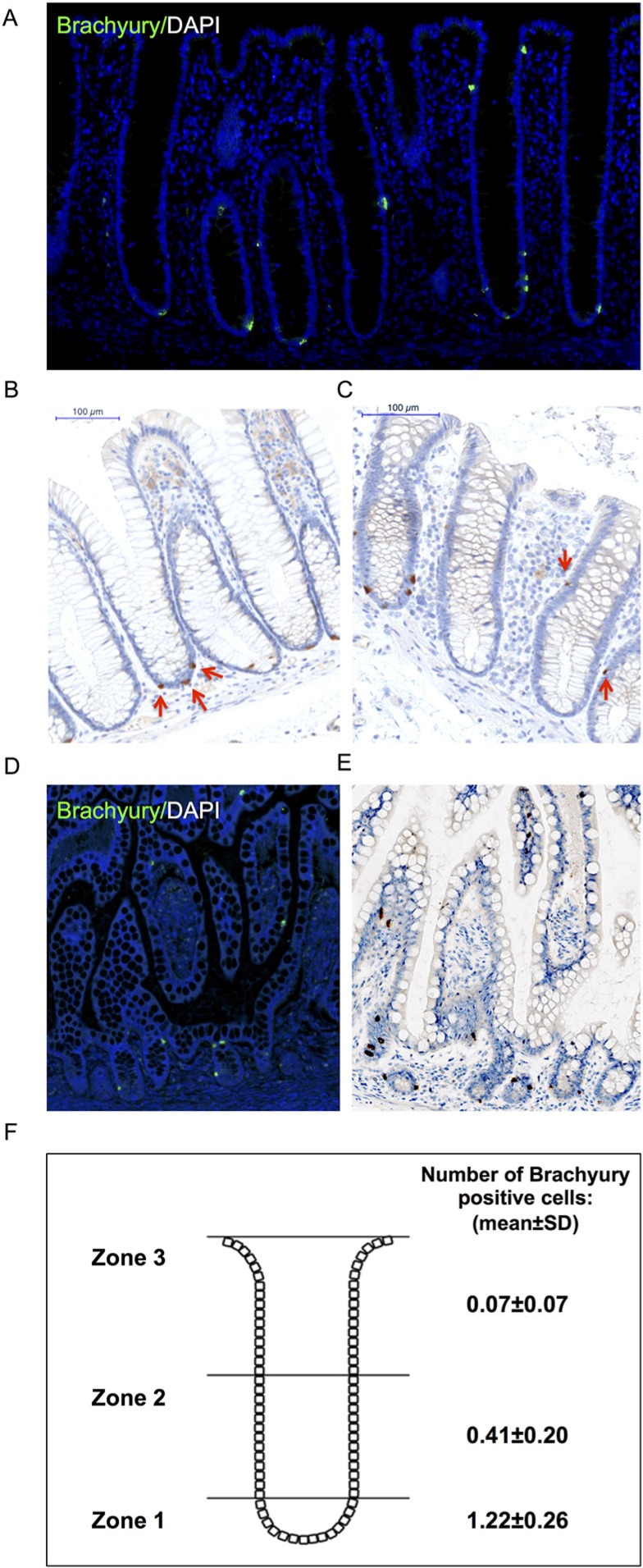
Immuno-detection of Brachyury in normal human intestinal crypts (**A**) Brachyury-IF on normal right-side colon FFPE sections; DAPI staining, blue; Brachyury staining green/ab140661. (**B, C**) Brachyury-IHC staining/ab140661 in normal human colon crypts (×20). Red arrows in B illustrate Brachyury-positive cells at crypt-base (consistent with SC zone), some crypts contain single Brachyury-positive cells in the TA zone (indicated by red arrows in (C). (**D**) Brachyury-IF: normal small intestinal FFPE sections. (**E**) Brachyury-IHC in normal human small intestine (×20). (**F**) Schematic diagram illustrating the mean number of Brachyury positive cells in three ‘zones’ per colonic crypt. Zone 1 encompasses the stem cell compartment, zone 2 encompasses the TA region and zone 3 encompasses the region containing differentiated cells (See Supplementary Figure 3 for classification of zones 1-3). The number of Brachyury positive cells in single crypts were scored and then a mean value calculated (and standard deviation) for each of the three zones. A total of 1930 pathologically normal crypts were counted from 22 independent patient derived samples (See SI for ‘normal’ tissue source information). FFPE: formalin-fixed paraffin-embedded.

**Figure 3:**
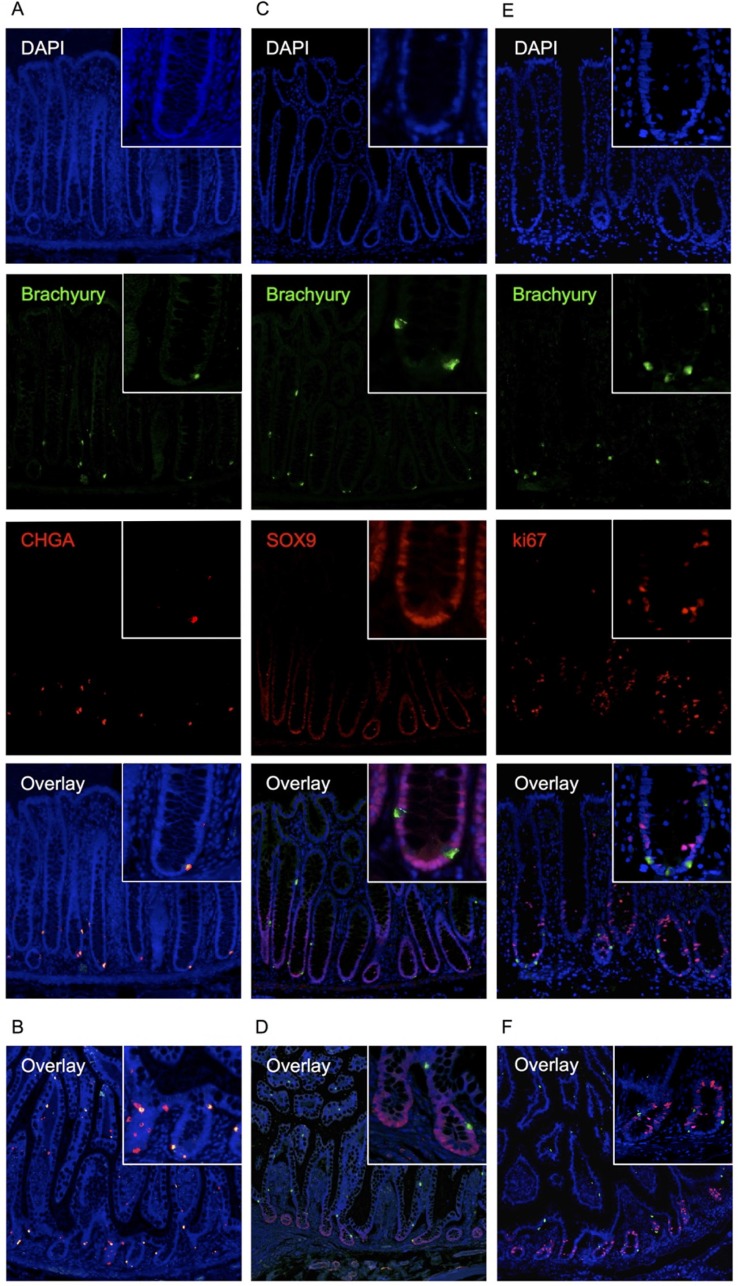
Immuno-detection of Brachyury in normal human intestinal crypts (**A**) co-IF for Brachyury/ChgA on normal colon crypt FFPE sections. Brachyury/ChgA-positive cells are located at the crypt-base (consistent with SC zone), some contain single Brachyury/ChgA-positive cells in the TA zone. (**B**) Brachyury/ChgA in the small intestine. (**C**) co-IF for Brachyury/SOX9 on normal colon crypt FFPE sections. (**D**) Brachyury/SOX9 in the small intestine. (**E**) co-IF for Brachyury/Ki67 (ab16667) in normal colon crypt FFPE sections. (**F**) Brachyury/Ki67 in the small intestine. For A-F, DAPI staining, blue; Brachyury staining, green/ab140661; ChgA/Sox9/Ki67 staining, red.

We explored this expression further by immuno-staining in human adult intestinal tissue. To this end, we validated the specificity of the anti-Brachyury monoclonal antibody (ab140661) using siRNA-mediated Brachyury-depletion in cancer cells (Supplementary Figure 2A, 2B). Using this antibody, we demonstrate that strong cytoplasmic Brachyury staining is present in a small cohort of cells within the normal human colonic crypt and small intestine (Figure 2A–2E; see also SI for source of ‘normal’ tissue). [Note: this strong cytoplasmic staining is very distinct from weaker cytoplasmic staining that we sometimes observed, and only strong staining is considered here]. Remarkably, most crypts contain at least one strongly Brachyury-positive cell in the SC compartment. Some crypts also contain Brachyury-positive cells in the TA zone of the crypt and more rarely towards the luminal surface (Figure 2F). [Similar results were obtained using another previously validated anti-Brachyury antibody (ab57480) [16], data not shown; and anti-Brachyury antibody (sc-20109) [23], data not shown]. Brachyury-positive cells had the morphology and location of EECs, that is, adjacent to the crypt-basement membrane with an abluminal disposition and located mainly at the crypt-base. Frequent overlap is observed between strongly stained Brachyury-positive cells and ChgA, a marker of differentiated EECs (Figure 3A, 3B). However, not all ChgA-positive cells are Brachyury-positive and it appears that Brachyury-positive cells are a distinct sub-population of EECs. Interestingly we observed that in the colon (Figure 3A), most ChgA-positive cells are Brachyury-positive whereas in the small intestine (Figure 3B) there are more ChgA-positive cells that are negative for Brachyury.

Cells that were positive for the post-mitotic EEC marker ChgA, were found to be negative for the nuclear localised proliferation marker Ki67 (Figure 3E, 3F), indicating that these cells are unlikely to be proliferating. [However, we noted a co-localisation between strong Brachyury positive cells and cytoplasmic localised Ki67 when we used an alternative Ki67 antibody, ab15580 (Supplementary Figure 4); although the significance of this cytoplasmic localisation is unclear].

Given that in the mouse, a population of differentiated EECs with regenerative capacity (ChgA-positive, Ki67-negative) express high levels of Sox9, we explored whether the Brachyury-positive EECs we have identified are similarly marked as Sox9-high. We found that Brachyury-positive cells in the crypt are Sox9-negative (or sub-low) (Figure 3C, 3D) so in this respect, are not similar to the Sox9-high EECs seen in mouse SI crypts [6, 7]. It is interesting to note however, that like Sox9, Brachyury can act in a dose-dependent manner [15] and this may be an important functional feature of such transcription factors in EECs.

### The association between Brachyury and ChgA is maintained in occasional cells in CRCs

**Figure 4:**
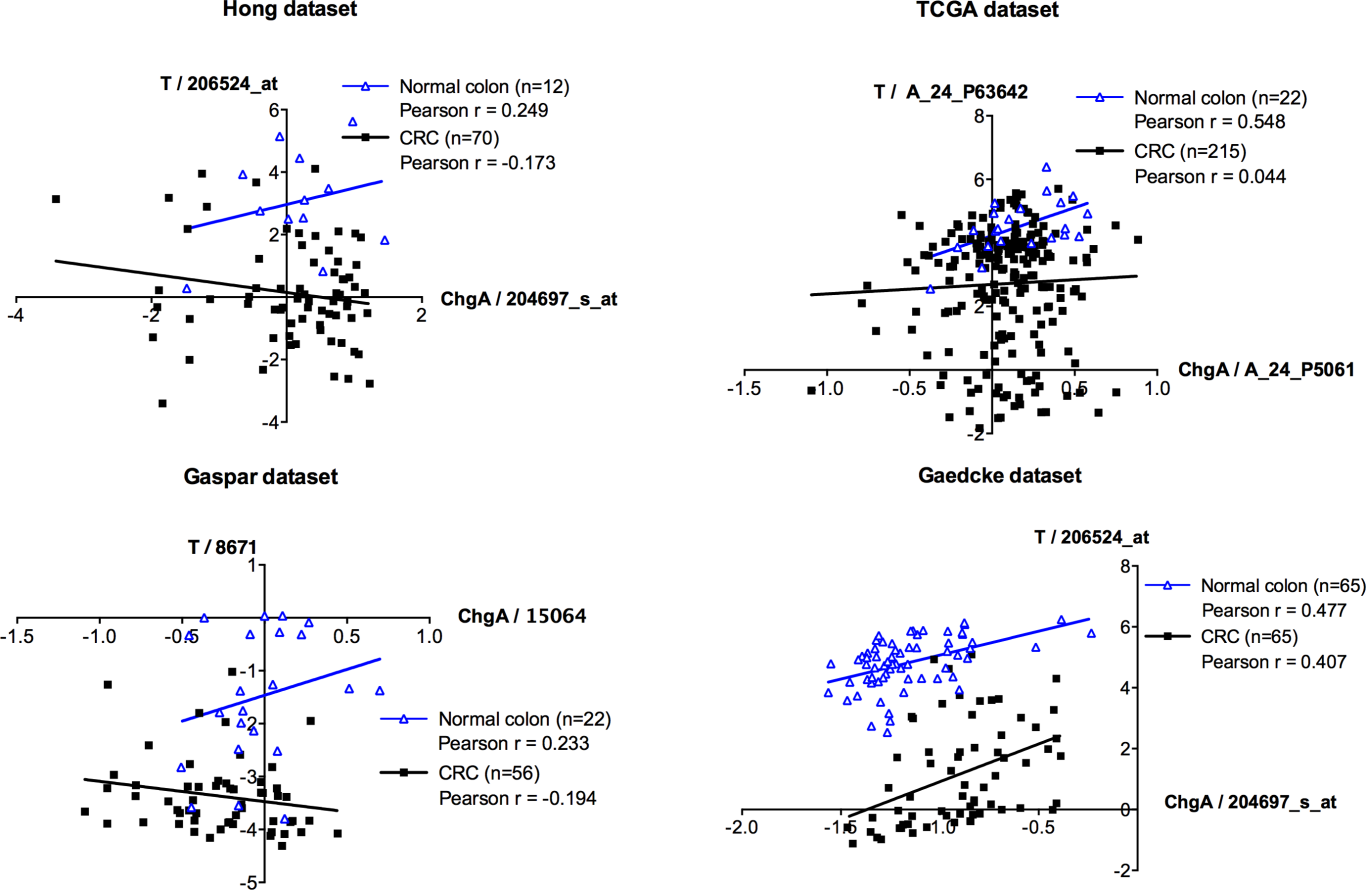
Microarray co-expression analyses between *ChgA* and *Brachyury* in normal and colorectal carcinoma tissues mRNA microarray expression for *Brachyury* (*T*) and *ChgA* were extracted from four independent datasets (Hong, TCGA, Gaspar and Gaedcke; See Supplementary Information 1) that have information for normal and colorectal cancer (CRC) samples. Pearson r correlation was used to evaluate the association between the expression levels of *Brachyury* and *ChgA* in the normal and in the CRC samples. *In silico* analysis indicates a direct correlation (Pearson *r* > 0.2) between *Brachyury* and *ChgA* expression in normal samples (blue line) for all datasets studied. In CRC samples (black line) no association was observed (−0.2 < Pearson *r* < 0.2) in three of the datasets (Hong, TCGA and Gaspar datasets) with the exception of the Gaedcke dataset that presents a direct association between *Brachyury* and *ChgA* (Pearson *r* > 0.2). Expression is present as log2 median-centered intensity values for each probe per study (original values extracted from Oncomine database). Each blue triangle represents a normal sample and each black square a CRC sample. ‘*n*’ represents the total number of samples in each dataset.

**Figure 5:**
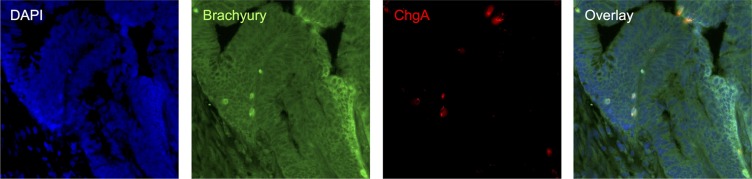
Immuno-detection of Brachyury and ChgA in poorly differentiated pT3N0 adenocarcinoma CRC tissue co-IF for Brachyury/ChgA on CRC FFPE section; DAPI labelled in blue, Brachyury labelled in green and ChgA in red. Strongly Brachyury stained cells are co-incident with cells being labelled with ChgA.

We then asked whether the association between Brachyury- and ChgA-positive cells was maintained in CRC patient derived material. Initially, we studied mRNA microarray data sets from four different sources (see Supplementary Information 1) to determine whether there was a correlation between *Brachyury* and *ChgA* expression in normal colon and in CRC tissues. A positive correlation between *Brachyury* and *ChgA* was observed in all data sets for normal colon (Figure 4), in accordance with our immuno-detection observations. However, we did not observe a positive correlation between *Brachyury* and *ChgA* in three out of the four data sets from CRC tissue (Figure 4). To resolve this further, co-IF was carried out on patient derived CRC material to determine the localisation patterns of Brachyury and ChgA within tumours. Three out of five of the cancers studied were Brachyury-positive but ChgA-negative (data not shown). Two tumours stained positively for both Brachyury and ChgA (example shown in Figure 5). Staining of Brachyury within the tumour is broad and heterogeneous with occasional cells that are dual stained for both Brachyury (strongly stained cells) and ChgA (Figure 5). It appears that the staining of Brachyury in the tumour is more diffuse than in normal tissue, suggesting broad functions of Brachyury in response to tumour-associated signals. This broader staining pattern for Brachyury in CRCs might explain why the association between Brachyury and ChgA observed earlier, is lost in the microarray analysis. Cells that are dual stained for Brachyury and ChgA are always strongly stained for Brachyury.

## DISCUSSION

In the present study, we have identified a previously uncharacterised sub-set of Brachyury and ChgA-positive EECs in adult human small intestinal and colonic crypts. Evidence is mounting to suggest that sub-groups of EECs might be involved in SC homeostasis and/or crypt regeneration. In support of this, recent RNA-seq/GO cluster analysis from our group suggests Brachyury is involved in somatic SC maintenance [16]. It is interesting to speculate that Brachyury is required for a sub-set of secretory lineage EECs in intestinal crypts that have plasticity and may modulate the activity of surrounding cells, possibly even acting as (reserve) SCs, similar to other known examples.

As mentioned previously, intestinal SCs have been shown to give rise to tumours [9, 10] and the link between SCs and CSCs has now been shown for many different tumour types, including CRC [24, 25]. We have shown previously that Brachyury is correlated with CRC cells that have CSC characteristics [18]; furthermore, Brachyury has been shown to be required for maintenance of spermatogonial SCs [20] demonstrating its importance in maintaining different somatic SC populations. Similar to the association of Brachyury with SCs, sub-populations of EECs are correlated with reserve SC characteristics, as described above. Taken together, it is possible that cells marked jointly by Brachyury and ChgA might represent a population of cells within the tumour that can demonstrate CSC properties which include tumour aggressiveness, drug resistance, renewal, EMT and invasion [25, 26].

ChgA signals have been linked to tumour aggressiveness in prostate cancer, where the presence of proteins/hormones produced from neuroendocrine cells may act as growth factors contributing to paracrine effects on surrounding stromal cells [27]. Furthermore, CRCs displaying endocrine differentiation have been reported to have a worse prognosis [13]. We showed that the association between Brachyury and ChgA marked cells continues beyond cells of the normal intestinal crypts and is seen also in CRC tissue. Here, Brachyury is more widely dispersed than ChgA, suggesting that Brachyury may have broader ranging functions in different regions of the tumour, possibly responding to regional tumour associated signals. However, given the link between Brachyury, SCs [20] and CSCs [18], and ChgA with SCs, it is possible that the Brachyury/ChgA-positive cells observed in regions of CRCs represent regions rich in CSCs.

There is mounting evidence that factors involved in regulating EMT are also involved in SC biology [28] and Brachyury has previously been shown to induce EMT in cancer [29]. Brachyury may serve as another example of such a regulator of EMT, possibly also then being linked to SCs in the normal crypt and CSCs; particularly since such factors seem to act in a dose-dependent manner such as Twist where low levels promote stemness and proliferation and high levels promote EMT [30].

The novel identification of a sub-class of ChgA-positive, Brachyury-positive EECs in adult, normal intestinal crypts and CRC cells provides an intriguing platform for further studies associated with normal homeostasis and cancer biology.

## MATERIALS AND METHODS

### Cell culture

Cell lines, growth conditions and details of authentication reports for cell lines used in this study are provided in Supplementary Information 1.

### Quantitative real-time PCR

cDNA was generated from the total RNA prepared from 16 normal human tissues using Quantitect Reverse Transcription kit (Qiagen, #205310). Real-time PCR reactions were carried out in triplicate on a CFX96 Real-Time System C1000 Thermal Cycler (BioRad) using Quantifast SYBR green RT-PCR kit (Qiagen, #204154). Primers used and further details are provided in Supplementary Information 1.

### Brachyury-knockdown

Brachyury siRNA (Qiagen, SI04133521, SI04144483) and negative control siRNA (Qiagen, 1027280) was used at a final concentration of 5 nM. Transfection was carried out with HiPerFect Reagent (Qiagen, 301705) according to the manufacturer's instructions. Cells were harvested 24 hours post-transfection. Details of *Brachyury* over-expression are provided in Supplementary Information 1.

### Western blotting

Western blotting protocols, antibodies and antibody dilutions used are described in detail in Supplementary Information 1.

### Immunohistochemistry (IHC) and immunofluorescence (IF)

Detailed protocols are provided in Supplementary Information 1, including tables of primary and secondary antibodies and relevant dilutions.

## SUPPLEMENTARY MATERIALS TABLES AND FIGURES


