# Supplementary material for: Brachyury identifies a class of enteroendocrine cells in normal human intestinal crypts and colorectal cancer
Source: Oncotarget. 2016 Feb 5;7(10):11478–86. doi: 10.18632/oncotarget.7202 (PMC4905487; doi:10.18632/oncotarget.7202)
Supplement: Supplementary file 1 [file oncotarget-07-11478-s001.pdf]

Brachyury identifies a class of enteroendocrine cells in normal human intestinal crypts and colorectal cancer

Supplementary Materials

MATERIALS AND METHODS

Western blot

Whole-cell lysates were prepared using M-PER lysis buffer (Thermo Scientific, #78503), Halt Protease Inhibitor Cocktail (Thermo Scientific, #87785) and Halt Phosphatase Inhibitor Cocktail (Thermo Scientific, #78420). Membranes were probed with primary antibodies in 5% dry milk/PBS/0.5% Tween 20. Incubation with secondary antibodies was performed at 4°C overnight, followed by three 5 minute washes in 5% dry milk/PBS/0.5% Tween 20 at room temperature. Incubation with corresponding secondary antibodies in appropriate dilutions was performed at room temperature for 1 hour, followed by a 10 minute wash in milk solution and 3 additional 10 minute washes in PBS/0.5% Tween 20. Antibody detection was performed using Pierce ECL Plus Western Blotting Substrate (Thermo Scientific, 32132). Antibodies used are listed in Table 1.

Quantitative real-time PCR

cDNA was generated from the total RNA prepared from 16 normal human tissues using Quantitect Reverse Transcription kit (Qiagen, #205310). Real-time PCR reactions were carried out in triplicate in total volume of 25 µl on a CFX96 Real-Time System C1000 Thermal Cycler (BioRad) using Quantifast SYBR green RT-PCR kit (Qiagen, #204154) with 1.5 µl of diluted cDNA template (equivalent to 7.5 ng RNA). Genes of interest were amplified according to the manufacturer›s directions (initial denaturation at 95°C for 5 min, 40 cycles of 95°C for 10 s and 60°C for 30 s), followed by a melting curve analysis. QuantiTect Primer Assay (Qiagen) was used for genes listed in Table 2. Sequences of the primers designed using Primer BLAST primer designing tool (NCBI) to detect Brachyury are listed in Table 3. CFX Manager™ Version 1.0 software (Bio-Rad) with default parameters was used to assess primer efficiency and specificity and to determine the threshold cycle (Ct) values. Results were normalized to a combination of two reference genes and the relative fold change in expression was computed by the ΔΔCt method.

Cell culture

SW480 cells (ECACC, cell line authentication report number 710418378) were cultured in DMEM medium (Life Technologies, #61965) supplemented with 10% fetal calf serum; H460 cells (ECACC, cell line authentication report number 710418378) were grown in RPMI medium (Life Technologies, #61870) supplemented with 10% fetal calf serum. Both cell lines have undergone 16 loci STR authentication (LGC Standards, UK).

Brachyury-knockdown

Brachyury siRNA (Qiagen, SI04133521, SI04144483) and negative control siRNA (Qiagen, 1027280) was used at a final concentration of 5 nM. Transfection was carried out with HiPerFect Reagent (Qiagen, 301705) according to the manufacturer's instructions. Cells were harvested 24 hours post-transfection.

Brachyury-overexpression

The protein-coding region of Brachyury was cloned into the eukaryotic expression vector, pFN21A HaloTag® CMV Flexi® Vector (Promega, G2831) containing an N-terminal HaloTag as described in the manufacturer's instructions. SW480 cells (2×10^6) were grown in T75 flasks. Cells were transfected 24 hours later with the HaloTag-Brachyury fusion construct (FuGENE HD transfection reagent (Promega, E2311) according to manufacturer's protocols. Briefly, the ratio of FuGENE HD reagent to plasmid DNA was 3:1 and 23 µg of plasmid DNA was used per flask. Cells were harvested 48 hours post-transfection.

Source of tissue samples

Patients undergoing colonic resection for colon cancer were used to obtain pathologically normal and colon cancer FFPE tissue samples. Areas of normal bowel (terminal ileum and/or right sided colon) distal to the primary tumour were sampled from the same pathological specimens. Written consent was obtained from individual patients and ethical approval was from the local research ethics committee (North Wales Research Ethics Committee–West; reference 12/WA/0042). 22 of these normal tissue samples were used to derive counts for Figure 2F.

5 patient derived CRC samples (3 colon and 2 rectal–samples samples from RICE trial–all cancers were sporadic, one sample was grade T3N1 and all others were T3N0) were used to study co-localization of Brachyury and ChgA by IF; an example is presented in Figure 5.

Supplementary Table S1: Primary and secondary antibodies for WB and their dilutions and sources

Primary Antibody	Clone no.	Cat. no.	Source	Host	Clonality	Western Blot dilution
Anti-Brachyury	1H9A2	ab140661	Abcam	Mouse	Monoclonal	1/500
Anti-GAPDH	F0911	sc-365062	Santa-Cruz	Mouse	Monoclonal	1/2000
Anti-α-Tubulin		T6074	Sigma	Mouse	Monoclonal	1/8000
**Secondary Antibody Cat. No.**	**Source**	**Host**	**Species reactivity**	**Isotype**	**Conjugate**	**Western Blot dilution**
7076	Cell signaling	Horse	Mouse	IgG	HRP	1/3000

Supplementary Table S2: QuantiTect Primer Assays (for use in real-time RT-PCR with SYBR Green detection)

Gene	QuantiTect Primer Assay Name	Catalogue number
GAPDH	Hs_GAPDH_2_SG	QT01192646
HSPCB	Hs_HSP90AB1_1_SG	QT01002624

Supplementary Table S3: Brachyury qRT-PCR primer sequences and their expected product size in base pairs (bp)

Gene	Primer Name	Primer Sequence	Product region	Product size (bp)
Brachyury	Bry Q Fw<br>Bry Q Rv	5′- GTGACAGGTACCCAACCCTG -3′<br>5′- GGTGAGTTGTCAGAATAGGTTGGA -3′	1346–1442	97 bp

Furthermore, the CRC samples that were positive for both Brachyury and ChgA (1 colon and 1 rectal) were used in Brachyury/ChgA quantification analysis (SI below). These cancers were poorly differentiated, T3N0 adenocarcinomas.

Immunohistochemistry (IHC)

The tissue samples were fixed in formalin and embedded in paraffin and IHC analysis was performed on 4 µm tissue sections. Staining was automated on a Ventana Benchmark XT machine using a standard immunohistochemistry protocol with a heat retrieval method. Antigen retrieval and antibody dilutions are listed in Table 4. 3, 3- Diaminobenzidine (DAB) was used as a chromogenic substrate, and the slides were counterstained using haematoxylin. Negative controls were omission of the primary antibody. Images were acquired on an Axio Scan. Z1 Digital Slide Scanner and ZEN software (Zeiss).

Indirect immunofluorescence

For immunostaining of tissue, 4 µm sections were deparaffinized and rehydrated by passing them three times through xylene, two times through 100% alcohol, and two times through 70% alcohol for 10 minutes each, followed by rinsing with distilled water twice. Antigen retrieval was performed using heat-induced epitope retrieval by maintaining slides at a sub-boiling temperature (98–100˚C) in 10 mM sodium citrate buffer pH 6.0 for 10 minutes. After rinsing with distilled water, the sections were incubated in 5% FBS/ 0.3% Triton™ X-100/1× PBS for 1 hour at room temperature to block nonspecific binding and permeabilize membranes. Next, the slides were stained overnight with primary antibodies (Table 5) diluted in 1% BSA/ 0.3% Triton™ X-100/1× PBS, followed by three washes with 1x PBS for 5 minutes each. After washing, the slides were incubated with Alexa Fluor secondary antibodies (Table 6) diluted in 1% BSA/ 0.3% Triton™ X-100/1× PBS for 2 hours at room temperature in the dark, followed by three washes with 1x PBS for 5 minutes each. Coverslips were mounted with Prolong® Gold Antifade Reagent with DAPI (Cell Signalling, #8961). Slides were analyzed using an Axio Scan. Z1 Digital Slide Scanner and ZEN software (Zeiss).

ImageJ analysis of co-staining in tissue samples

In order to quantify the percentage of CRC tissue that was co-stained for both Brachyury and ChgA a region of interest (ROI) was firstly identified from the whole tissue image (selected to avoid edge effect). This was then assessed for staining of each protein by setting a threshold using the thresholding tool in ImageJ and quantifying in terms of percentage of cells above the threshold in the ROI (according to Quantitative analysis of histological staining

and fluorescence using ImageJ http://onlinelibrary.wiley.com/doi/10.1002/ar.22641/full)

Using this approach, the two CRCs that were Brachyury/CgA positive gave the following:

CRC 1 (poorly differentiated pT3N0 adenocarcinoma of the ascending colon): 32% Brachyury; 0.74% ChgA

CRC2 (poorly differentiated pT3N0 adenocarcinoma of rectum): 10% Brachyury; 0.17% ChgA

Note: Cells that were ChgA positive in the cancer samples were always Brachyury positive, so 100% of ChgA cells are also Brachyury positive but only 2.3% and 1.7% of Brachyury positive cells are also ChgA positive.

Oncomine microarray data analysis

Brachyury and ChgA mRNA expression values were extracted from the Oncomine database https://www.oncomine.com/resource/main.html (Rhodes DR, Kalyana-Sundaram S, Mahavisno V, Varambally R, Yu J, Briggs BB. Et al. Oncomine 3.0: Genes, pathways, and networks in a collection of 18,000 cancer gene expression profiles. Neoplasia 2007: 9:166–80). Four independent colorectal cancer datasets-TCGA (Cancer Genome Atlas Network. Comprehensive molecular characterization of human colon and rectal cancer. Nature 2012/07/18), Gaedcke (Gaedcke J, Grade M, Jung K, Camps J, Jo P, Emons G, Gehoff A, Sax U, Schirmer M, Becker H, Beissbarth T, Ried T, Ghadimi BM. Mutated KRAS results in overexpression of DUSP4, a MAP-kinase phosphatase, and SMYD3, a histone methyltransferase, in rectal carcinomas. Genes Chromosomes Cancer 2010/11/01), Gaspar (Gaspar C, Cardoso J, Franken P, Molenaar L, Morreau H, Moslein G, Sampson J, Boer JM, de Menezes RX, Fodde R. Cross-species comparison of human and mouse intestinal polyps reveals conserved mechanisms in adenomatous polyposis coli (APC)-driven tumorigenesis. Am J Pathol 2008/05/01) and Hong (Hong Y, Downey T, Eu KW, Koh PK, Cheah PY. A ‘metastasis-prone’ signature for early-stage mismatch-repair proficient sporadic colorectal cancer patients and its implications for possible therapeutics. Clin Exp Metastasis 2010/02/01) - were assessed, all with information both for normal (total n = 121) and colorectal cancer (total n = 406) tissues. Pearson test was used to evaluate the correlation between gene expression profiles. The statistical analysis was performed using Prism GraphPad software (version 5.0a).

Supplementary Table S4: Primary antibody for IHC, dilution, pre-treatment, and source

Antibody	Clone	Host	Dilution	Antigen retrieval/Protocol	Source	Cat. number
Anti-Brachyury	Monoclonal	Mouse	1:50–1:300	Heat 76ºC (4′), 95ºC (8′), 100ºC (4′) + cell conditioning (CC1*) 1°ab 1 hr + Ultra Wash	Abcam	ab140661

*CC1 is a Ventana product (Ventana Medical Systems Inc, Tucson, AZ).

Supplementary Table S5: Primary antibodies for IF and their dilutions and sources

Antibody	Clone	Host	Dilution	Source	Cat. number
Anti-Brachyury	Monoclonal	Mouse	1:100	Abcam	ab140661
Anti-Brachyury	Monoclonal	Mouse	1:100	Abcam	ab57480
Anti-Chromogranin A	Polyclonal	Rabbit	1:100	Abcam	ab15160
Anti-SOX9	Monoclonal	Rabbit	1:100	Abcam	ab185230
Anti-Ki67	Monoclonal	Rabbit	1:100	Abcam	ab16667
Anti-Ki67	Polyclonal	Rabbit	1:100	Abcam	ab15580

Supplementary Table S6: Secondary antibodies for IF and their dilutions and sources

Antibody	Host	Species Reactivity	Dye/Label	Excitation/ Emision	Dilution	Source	Cat. Number
Anti-mouse IgG (H + L)	Goat	Mouse	Alexa Fluor® 488	495/519	1:500	Life Technologies	A11029
Anti-mouse IgG (H + L)	Goat	Mouse	Alexa Fluor® 568	578/603	1:500	Life Technologies	A11031
Anti-goat IgG (H + L)	Donkey	Goat	Alexa Fluor® 488	495/519	1:500	Life Technologies	A11055
Anti-goat IgG (H + L)	Donkey	Goat	Alexa Fluor® 568	578/603	1:500	Life Technologies	A11057
Anti-rabbit IgG (H + L)	Goat	Rabbit	Alexa Fluor® 488	495/519	1:500	Life Technologies	A11034
Anti-rabbit IgG (H + L)	Goat	Rabbit	Alexa Fluor® 568	578/603	1:500	Life Technologies	A11011

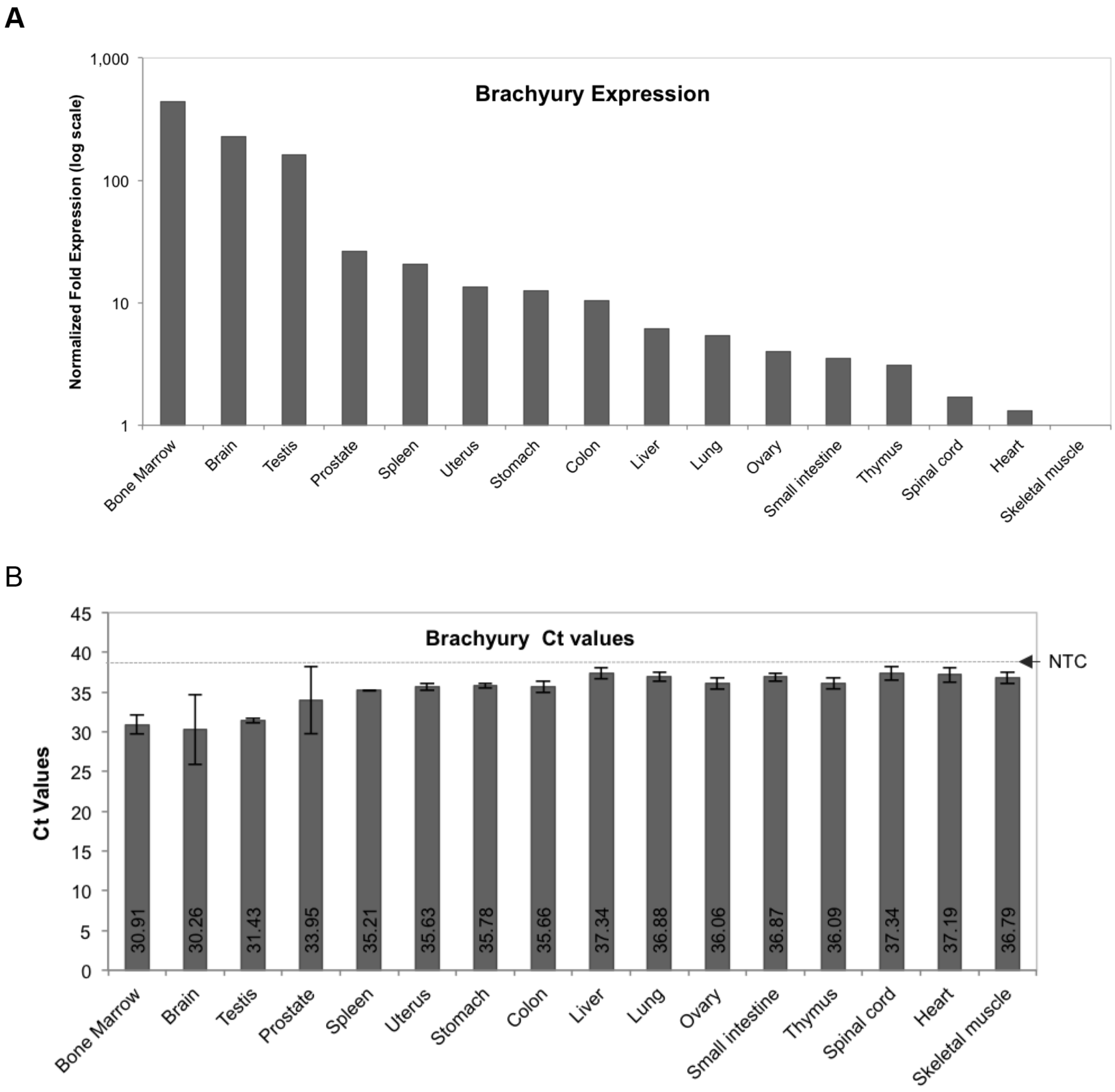

C

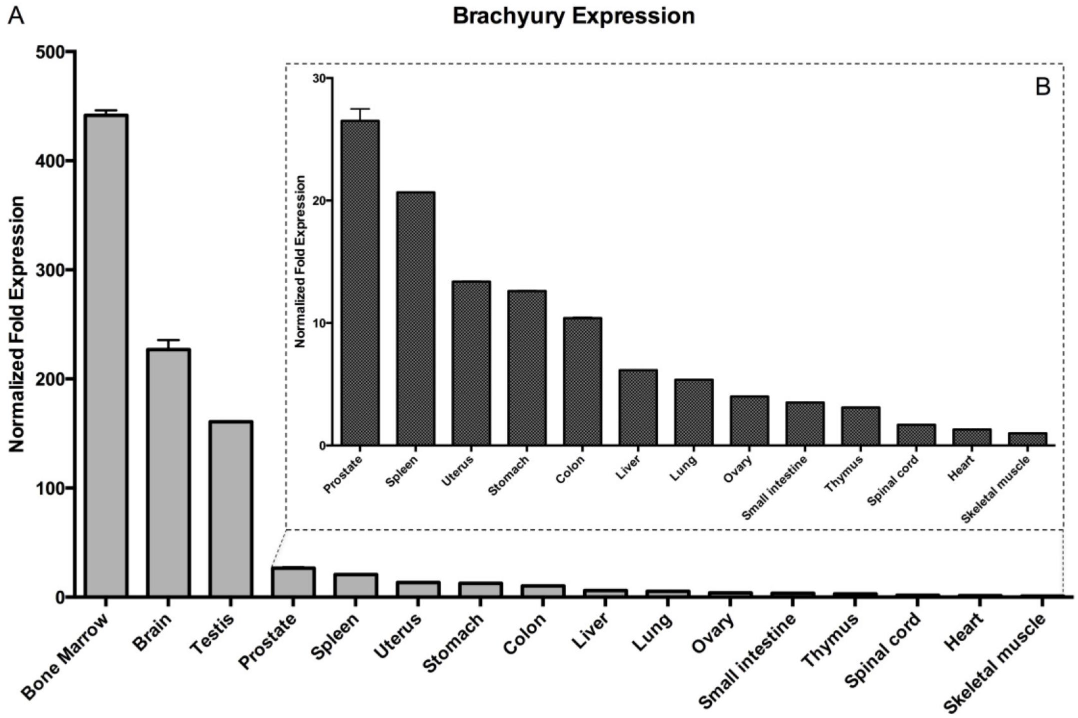

Supplementary Figure S1: qRT PCR analysis showing expression levels of *Brachyury* in a range of normal tissues. cDNA was generated from the total RNA prepared from 16 normal human tissues (obtained post-mortem). (**A**) Relative *Brachyury* expression. Results were normalized to a combination of two endogenous reference genes (*GAPDH* and *HSPCB*) and the relative fold change in expression was computed by the ΔΔCt method. Error bars show standard error of the mean, the y axis scale is logarithmic. (**B**) Results showing cycle threshold (Ct) values of Brachyury and NTC (no-template control, Ct = 38), the axis scale is linear. (**C**) The inset figure (B) is derived from the outer part 'A' and shows the normalised expression data with the three highest expressing tissues removed in order to see relative expression levels in low expressing tissues on a linear scale. In all graphs, 'Fold Expression' represents change relative to lowest value observed.

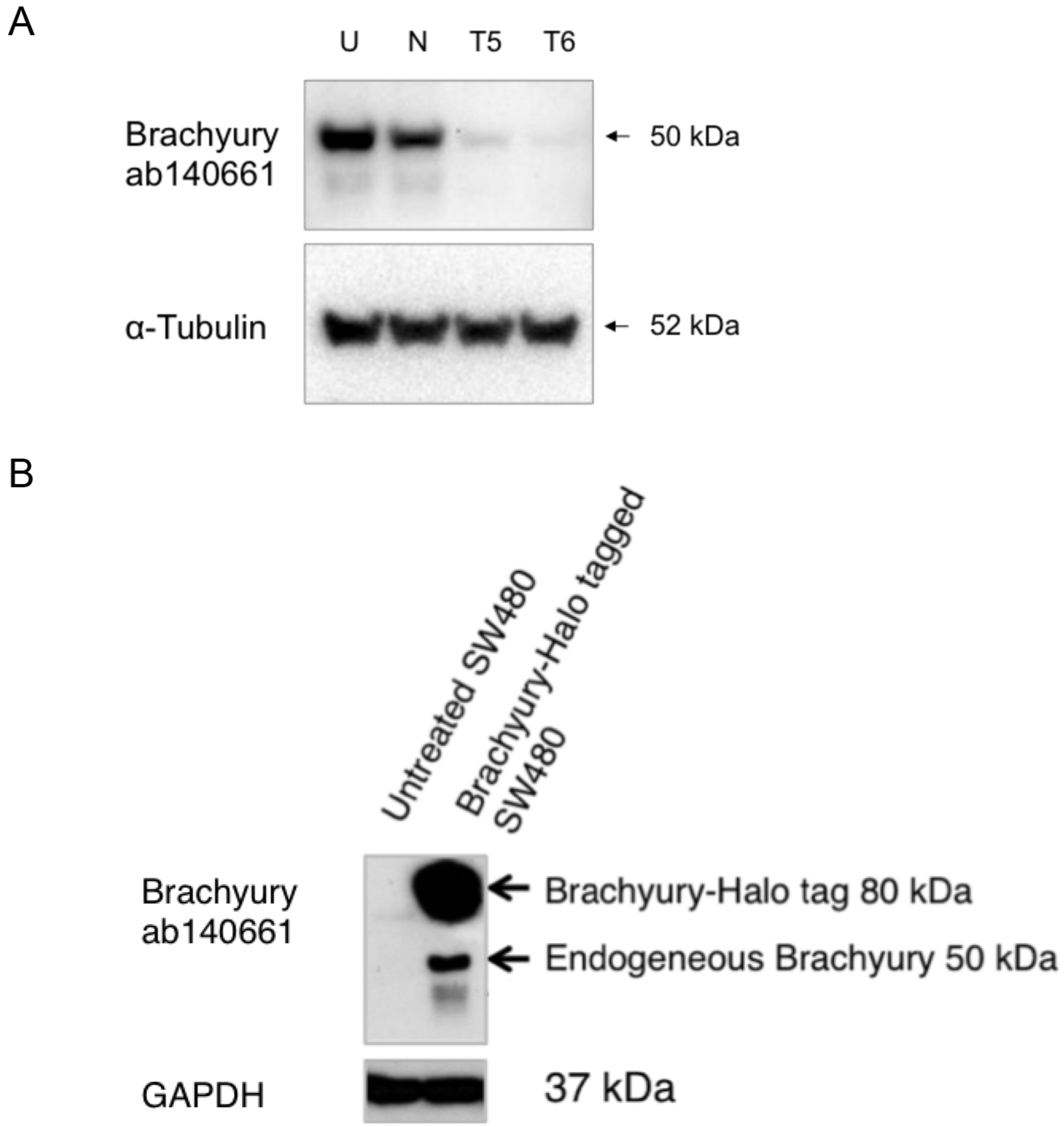

Supplementary Figure S2: Brachyury antibody validation for immunohistochemistry and immunofluorescence. (**A**) siRNA depletion of Brachyury and western blot using the mouse monoclonal antibody from Abcam (ab140661). U–untreated H460 cells, N–negative control siRNA treated H460 cells, T5–Brachyury siRNA (Hs_T_5) treated H460 cells, T6–Brachyury siRNA (Hs_T_6) treated H460 cells, loading was controlled by α-Tubulin. Western blot images were cropped for size. (**B**) Detection of bands by the mouse monoclonal antibody from Abcam (ab140661) on western blot from Brachyury-Halo tag overexpression in SW480, loading was controlled by GAPDH.

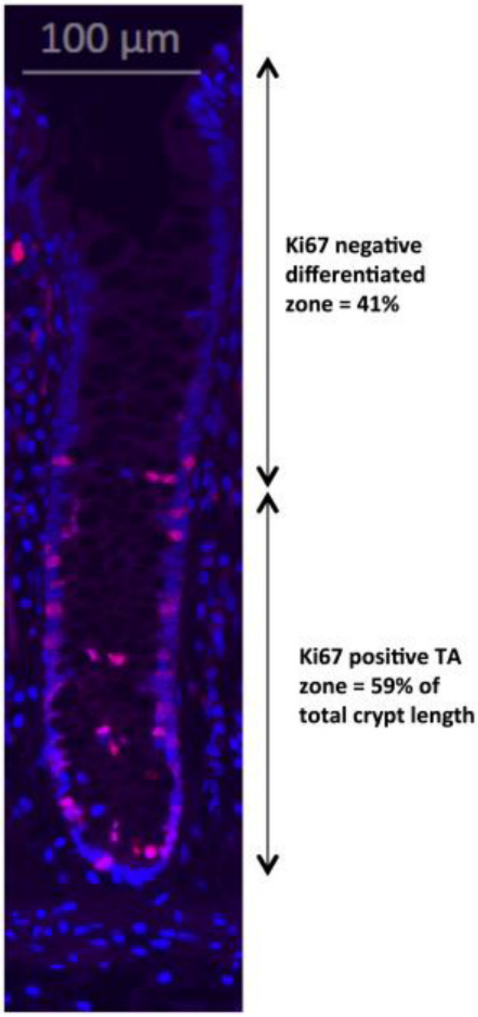

Supplementary Figure S3: Use of the proliferative marker Ki67 to define the zones of the normal colon crypts.

A total of 25 crypts stained with Ki67 (red) from one patient were used to define the transit amplifying zone (zone 2) and the differentiated zone (zone 3) of the crypts.
The stem cell zone (zone 1) was defined as +5 cells from the bottom of the crypt.
The average crypt length was 358.6 µm.
The average length of the Ki67 positive region was 210.4 µm (59%).
We defined this region (minus the stem cell zone) as the transit amplifying zone, zone 2.
The average length of the Ki67 negative region was 148.4 µm (41%).
We defined this as the differentiated zone, zone 3.

Pairwise comparison for differences in the frequency of Brachyury positive cells in zone 1, 2 and 3:

Group 1	Group 2	*P* value
**Zone 1**	Zone 2	< 0.0001
**Zone 1**	Zone 3	< 0.0001
**Zone 3**	Zone 2	< 0.0001

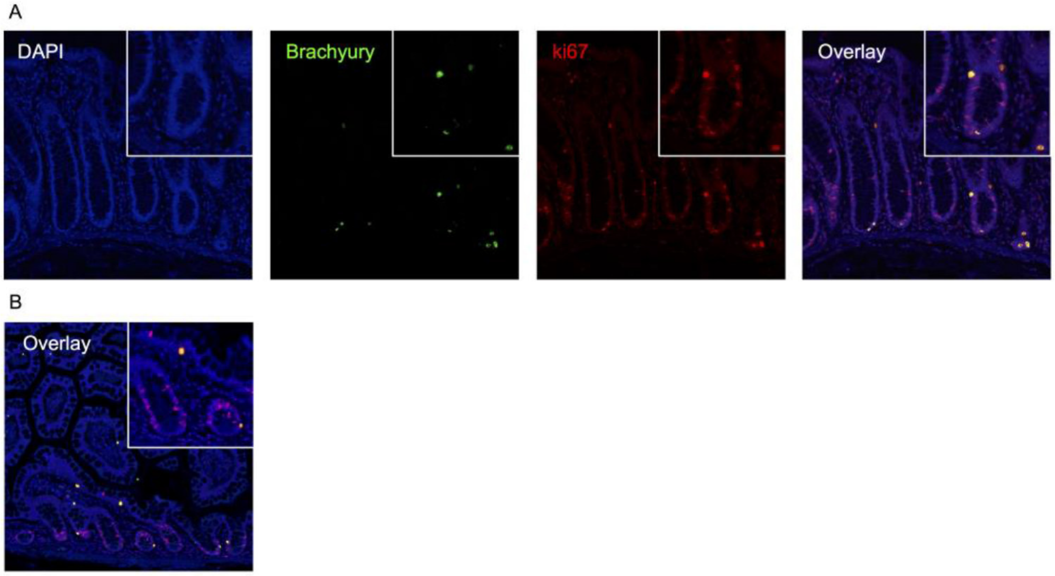

Supplementary Figure S4: Immuno-detection of Brachyury in normal human intestinal crypts. (**A**) co-IF for Brachyury and Ki67 (antibody 15580) in normal colon FFPE sections. (**B**) Brachyury/Ki67 in normal small intestine. For A and B, DAPI staining, blue; Brachyury staining, green/ab140661; Ki67 staining, red/ab15580.
